# Diagnostic Pitfalls of Oligodendroglioma-like Morphology: Integrated Reappraisal of 23 Non-Oligodendroglial Central Nervous System Tumors

**DOI:** 10.3390/diagnostics16172723

**Published:** 2026-08-26

**Authors:** Efe Yetişgin, Nazlı Türk, Gökhan Veli Alkan, Evrim Önder

**Affiliations:** 1 Department of Pathology, Ankara Etlik City Hospital, 06170 Ankara, Türkiye; 2 Department of Pathology, Ordu Training and Research Hospital, Ordu University, 52200 Ordu, Türkiye

**Keywords:** oligodendroglioma-like morphology, diagnostic pitfall, integrated diagnosis, central nervous system tumors, immunohistochemistry, molecular pathology, next-generation sequencing, neuropathology

## Abstract

**Background/Objectives:** Oligodendroglioma-like morphology may generate diagnostic uncertainty in central nervous system tumors, although oligodendroglioma is defined by the integrated presence of IDH mutation and whole-arm 1p/19q codeletion. This study aimed to evaluate the diagnostic pitfalls of oligodendroglioma-like morphology by comparing initial microscopic impressions with final integrated diagnoses. **Methods:** We retrospectively reviewed 23 non-oligodendroglial central nervous system tumors showing focal or predominant oligodendroglioma-like morphology but not fulfilling the integrated diagnostic criteria for oligodendroglioma. Clinical, radiological, histopathological, immunohistochemical, molecular/cytogenetic, initial microscopic impression, final integrated diagnosis, and follow-up data were analyzed descriptively. **Results:** Initial microscopic impressions included low-grade glial tumor, high-grade glial tumor, dysembryoplastic neuroepithelial tumor, ependymoma, astroblastoma, oligodendroglioma, dysplasia, central neurocytoma, metastatic epithelial tumor, and pilocytic astrocytoma/low-grade glial tumor. Final integrated diagnoses were astrocytoma, IDH-mutant, CNS WHO grade 2–4 (*n* = 9); dysembryoplastic neuroepithelial tumor (*n* = 3); central neurocytoma (*n* = 3); metastatic renal cell carcinoma (*n* = 2); low-grade glioneuronal tumor (*n* = 2); and PLNTY, glioblastoma IDH-wildtype, supratentorial ependymoma, and pilocytic astrocytoma (*n* = 1 each). **Conclusions:** Oligodendroglioma-like morphology should be interpreted as a diagnostic pitfall pattern rather than a specific diagnosis. Structured integrated reappraisal using targeted immunohistochemical and molecular/cytogenetic testing helps refine differential diagnosis in routine neuropathology practice.

## 1. Introduction

Oligodendroglioma has historically been recognized by a characteristic histopathological appearance, including uniform round nuclei, perinuclear clearing, delicate branching capillary networks, and frequent calcifications. However, in the current molecular era, these morphological features are no longer sufficient for diagnosis. The incorporation of molecular markers into central nervous system tumor classification has substantially improved diagnostic precision, particularly for diffuse gliomas and other molecularly defined entities [1,2,3,4,5]. According to the 2021 World Health Organization Classification of Tumors of the Central Nervous System, oligodendroglioma is defined by the combined presence of an IDH mutation and whole-arm 1p/19q codeletion [1]. Therefore, tumors with oligodendroglioma-like morphology but lacking these defining molecular alterations should be carefully evaluated for alternative diagnoses. Recent EANO recommendations further emphasize that molecular diagnostic tools are central to the practical implementation of WHO CNS5 criteria for gliomas, glioneuronal tumors, and neuronal tumors [5].

Oligodendroglioma-like morphology may be encountered in a broad spectrum of non-oligodendroglial central nervous system tumors, either as a predominant pattern or as a focal component within an otherwise more characteristic tumor architecture. Diffuse astrocytomas may show areas with small round cells and perinuclear clearing and may be distinguished from oligodendroglioma by the absence of 1p/19q codeletion, together with supportive findings such as *ATRX* loss and p53 accumulation [1,2,3,4,6]. In addition, cIMPACT-NOW recommendations and subsequent WHO CNS5 criteria have emphasized that selected molecular alterations can define clinically relevant glioma categories even when histological features are limited or heterogeneous, further underscoring the need for integrated histomolecular interpretation [1,3].

Several low-grade glioneuronal and epilepsy-associated tumors may also enter the differential diagnosis of oligodendroglioma. Dysembryoplastic neuroepithelial tumors may contain oligodendroglial-like cells, microcystic architecture, and neuronal elements, particularly in cortically based lesions associated with epilepsy [7,8]. Polymorphous low-grade neuroepithelial tumor of the young is another important mimic, characterized by oligodendroglioma-like components, frequent calcification, aberrant CD34 expression, and MAPK pathway alterations [9,10]. Circumscribed MAPK pathway-altered gliomas, including pilocytic astrocytoma, may occasionally show focal oligodendroglioma-like areas with monotonous round nuclei, perinuclear halos, and calcification, especially when classic biphasic architecture, Rosenthal fibers, and eosinophilic granular bodies are limited or underrepresented in small tissue samples [11,12,13,14,15]. Recent reviews of pediatric-type low-grade gliomas and glioneuronal tumors highlight the expanding molecular spectrum of these entities and the need for integrated interpretation in diagnostically overlapping cases [15].

Beyond diffuse gliomas and low-grade glial or glioneuronal tumors, other entities may also simulate oligodendroglioma on routine hematoxylin and eosin sections. Central neurocytoma may show uniform round cells with perinuclear halos and a delicate capillary network, particularly in intraventricular lesions [16]. Supratentorial clear cell ependymoma may mimic oligodendroglioma because of its clear-cell morphology, small round cells, calcification, and delicate vasculature, while recognition of ependymal rosettes/pseudorosettes and supportive molecular alterations may redirect the diagnosis [17,18,19,20]. Metastatic clear cell renal cell carcinoma may also show clear cytoplasm, perinuclear clearing, and rich capillary vasculature, creating a potential pitfall in limited biopsy material or in patients without an initially known renal primary [19].

This morphological overlap has practical diagnostic implications. In routine neuropathology practice, oligodendroglioma-like features may generate a broad range of initial microscopic impressions, including low-grade glial tumor, high-grade glial tumor, DNET, ependymoma, astroblastoma, oligodendroglioma, metastatic epithelial tumor, or other low-grade lesions. These initial impressions are not necessarily definitive diagnostic errors, but they reflect the real-world uncertainty created by overlapping morphology. Because oligodendroglioma, astrocytoma, glioblastoma, glioneuronal tumors, circumscribed gliomas, ependymoma, neuronal tumors, and metastatic tumors differ substantially in prognosis, treatment approach, and follow-up strategy, accurate integrated classification is clinically relevant [1,2,3,7,8,9,10,11,12,13,14,16,17,18,19,20,21,22,23].

Although individual oligodendroglioma mimics have been described in the literature, fewer studies have focused on the diagnostic process generated by oligodendroglioma-like morphology in routine practice. In particular, limited data are available on how initial microscopic impressions are refined or redirected by immunohistochemistry, fluorescence in situ hybridization, and next-generation sequencing in a heterogeneous cohort of non-oligodendroglial tumors. In the present study, we retrospectively analyzed 23 non-oligodendroglial central nervous system tumors showing focal or predominant oligodendroglioma-like morphology but not fulfilling the integrated diagnosis of oligodendroglioma. We aimed to characterize their clinicopathological, immunohistochemical, molecular/cytogenetic, and follow-up features; compare initial microscopic impressions with final integrated diagnoses; and identify the ancillary diagnostic clues that support accurate reclassification in routine neuropathology practice. We do not aim to re-establish the already accepted principle that integrated histomolecular diagnosis is required for CNS tumors. Instead, this study focuses on oligodendroglioma-like morphology as a recurring diagnostic pitfall pattern and maps the real-world diagnostic trajectory from initial microscopic impression to final integrated diagnosis.

## 2. Materials and Methods

### 2.1. Study Design and Case Selection

This study was designed as a single-center, retrospective, case-based diagnostic reappraisal study rather than a hypothesis-driven comparative diagnostic accuracy study. The archives of the Department of Pathology, Ankara Etlik City Hospital, were searched for central nervous system tumors diagnosed between 2022 and 2026 that showed focal or predominant oligodendroglioma-like histomorphological features but did not fulfill the integrated diagnostic criteria for oligodendroglioma.

Oligodendroglioma-like morphology was defined by the presence of one or more of the following features: small monotonous round nuclei, perinuclear clearing or halo formation, delicate branching or chicken-wire-like capillary vasculature, calcification, microcystic background, and/or a histological pattern raising the differential diagnosis of oligodendroglioma during routine microscopic evaluation. Cases were eligible for inclusion if sufficient hematoxylin and eosin-stained sections and/or formalin-fixed paraffin-embedded tissue blocks were available for review, and if the final integrated diagnosis was other than oligodendroglioma, IDH-mutant and 1p/19q-codeleted.

Cases with a final integrated diagnosis of oligodendroglioma, IDH-mutant and 1p/19q-codeleted were excluded. Additional exclusion criteria were insufficient tissue for histopathological review, lack of essential clinicopathological data, and absence of evaluable oligodendroglioma-like histomorphological features.

A total of 23 non-oligodendroglial central nervous system tumors fulfilled the inclusion criteria and were included in the final cohort.

### 2.2. Clinical and Radiological Data

Clinical and demographic data were obtained from electronic medical records, pathology reports, and available clinical documentation. The recorded parameters included age at diagnosis, sex, tumor location, available presenting clinical information, type of surgical procedure, postoperative follow-up status, residual or recurrent disease, and survival status.

Preoperative radiological descriptions were retrieved from available radiology reports and clinical records. Radiological variables included tumor location, cortical or subcortical involvement, intraventricular localization, cystic or necrotic components, calcification, contrast enhancement pattern, edema, hemorrhagic features, and preoperative radiological impression when available.

### 2.3. Histopathological Evaluation

All available hematoxylin and eosin-stained slides were retrospectively reviewed. Histopathological evaluation focused on the presence and distribution of oligodendroglioma-like features, including small monotonous round nuclei, perinuclear clearing or halo formation, delicate chicken-wire-like capillary vasculature, calcification, microcystic background, cortical infiltrative pattern, clear-cell-like morphology, rosette or pseudorosette formation, atypical neuronal elements, necrosis, microvascular proliferation, and mitotic activity.

For each case, the initial microscopic impression recorded during the diagnostic process was documented. These initial impressions included categories such as low-grade glial tumor, high-grade glial tumor, dysembryoplastic neuroepithelial tumor, ependymoma, astroblastoma, oligodendroglioma, central neurocytoma, dysplasia, metastatic epithelial tumor, and pilocytic astrocytoma/low-grade glial tumor. The final integrated diagnosis was then compared with the initial microscopic impression to evaluate diagnostic refinement or reclassification.

Final diagnoses were assigned according to the current integrated diagnostic approach for central nervous system tumors, incorporating histopathological, immunohistochemical, molecular, cytogenetic, and clinicoradiological findings when available.

### 2.4. Immunohistochemistry

Immunohistochemical findings were retrieved from pathology reports and, when necessary, reviewed on available stained slides. The antibody panel varied according to the original diagnostic work-up of each case. Evaluated markers included, when available, IDH1 R132H, *ATRX*, p53, Olig2, GFAP, synaptophysin, NeuN, CD34, EMA, Ki-67, PAX8, PAX2, CD10, SOX10, vimentin, D2-40, S100, TTF1, and other markers used in the original diagnostic evaluation.

IDH1 R132H positivity was interpreted as cytoplasmic and/or nuclear staining in tumor cells. *ATRX* loss was defined as loss of nuclear expression in tumor cells with retained internal positive control staining in non-neoplastic cells. p53 expression was assessed according to the observed nuclear staining pattern. Ki-67 proliferative index was recorded as the approximate percentage of positively stained tumor nuclei in the area of highest labeling.

The diagnostic contribution of immunohistochemistry was assessed descriptively according to whether the marker profile supported astrocytic, glioneuronal, neuronal, ependymal, circumscribed glioma, or metastatic epithelial/renal differentiation.

### 2.5. Molecular and Cytogenetic Analysis

Molecular and cytogenetic findings were interpreted in the context of the WHO CNS5 framework and recent EANO recommendations for molecular diagnostic testing in CNS tumors [1,5]. These included next-generation sequencing results, IDH mutation status, *TERT* promoter mutation status, *BRAF* alterations including *KIAA1549::BRAF* fusion, *PRKCA*-related alterations or fusions, *ZFTA*-related alterations, and other detected mutations, fusions, or copy number changes when available. Fluorescence in situ hybridization results for 1p/19q codeletion were also recorded when performed.

Cases with IDH mutation but without 1p/19q codeletion were classified within the astrocytic lineage when supported by histopathological and immunohistochemical findings. Cases without IDH mutation and without 1p/19q codeletion were evaluated in the context of their histological architecture, immunophenotype, anatomical location, and additional molecular alterations, including MAPK pathway alterations, glioneuronal tumor-associated alterations, ependymoma-associated alterations, and metastatic tumor-related diagnostic markers. The absence of pathogenic alterations by next-generation sequencing was recorded when no clinically relevant alteration was detected in the performed panel.

*CIC* and *FUBP1* alterations were not used as primary diagnostic criteria to establish or exclude oligodendroglioma in this cohort. According to WHO CNS5, the defining molecular criteria for oligodendroglioma are the combined presence of an IDH mutation and whole-arm 1p/19q codeletion. Therefore, tumors lacking this required integrated molecular profile were not classified as oligodendroglioma, even when oligodendroglioma-like morphology was present. Because this was a retrospective real-world diagnostic reappraisal study, *CIC* and *FUBP1* testing was not systematically available across all cases and was not included as a formal variable in the analysis.

Genome-wide DNA methylation profiling was not performed systematically in this cohort; therefore, methylation class results were not available for all 23 tumors and were not included in the formal analysis.

### 2.6. Initial Impression and Diagnostic Reclassification Analysis

To evaluate the diagnostic impact of oligodendroglioma-like morphology in routine practice, the initial microscopic impression of each case was compared with the final integrated diagnosis. This analysis was intended to document the spectrum of preliminary diagnostic considerations generated by oligodendroglioma-like morphology and to identify cases in which immunohistochemical, cytogenetic, and/or molecular findings redirected or refined the diagnosis.

Diagnostic reclassification was defined as a change from the initial microscopic impression to a different final integrated diagnostic category after incorporation of ancillary studies and clinicoradiological context. Diagnostic refinement was defined as further specification of a broad initial impression, such as low-grade glial tumor or high-grade glial tumor, into a precise integrated diagnosis.

### 2.7. Assessment of Ancillary Diagnostic Contribution

The contribution of ancillary testing was evaluated descriptively. For each diagnostic group, the immunohistochemical, cytogenetic, or molecular findings that supported final classification were recorded. The evaluated diagnostic contributions included IDH mutation and 1p/19q status for excluding true oligodendroglioma and supporting astrocytic lineage; *ATRX* and p53 patterns for astrocytic differentiation; *TERT* promoter mutation for molecularly defined glioblastoma; synaptophysin and NeuN for neuronal or glioneuronal differentiation; CD34 for PLNTY; GFAP, EMA, and *ZFTA* alteration for supratentorial ependymoma; PAX8, PAX2, CD10, vimentin, and epithelial marker expression for metastatic renal cell carcinoma; and *KIAA1549::BRAF* fusion for pilocytic astrocytoma.

This analysis was not designed as a formal diagnostic performance study and did not calculate sensitivity, specificity, or predictive values. Instead, the aim was to describe how ancillary findings contributed to diagnostic refinement in a real-world retrospective neuropathology cohort.

### 2.8. Diagnostic Grouping

For descriptive analysis, cases were grouped according to their final integrated diagnosis and broader diagnostic category. The main diagnostic categories were defined as glial tumors, glioneuronal tumors, neuronal tumors, ependymal tumors, and metastatic tumors. Circumscribed gliomas were included within the broader glial tumor category.

The final integrated diagnostic groups included diffuse astrocytoma, IDH-mutant; glioblastoma, IDH-wildtype, CNS WHO grade 4, molecularly defined by *TERT* promoter mutation; pilocytic astrocytoma, *KIAA1549::BRAF* fusion-positive; dysembryoplastic neuroepithelial tumor; low-grade glioneuronal tumor; polymorphous low-grade neuroepithelial tumor of the young; central neurocytoma; supratentorial ependymoma; and metastatic renal cell carcinoma.

### 2.9. Follow-Up Data

Follow-up data were obtained from electronic medical records when available. The recorded follow-up parameters included duration of follow-up, postoperative residual disease, recurrence or progression, and death during follow-up. Follow-up status was categorized as no residual or recurrent disease, recurrent disease, or death during follow-up.

### 2.10. Statistical Analysis

Statistical analyses were performed using IBM SPSS Statistics version 28.0. Categorical variables were summarized as numbers and percentages. Continuous variables were expressed as median and range. Because of the retrospective design, limited cohort size, and heterogeneous diagnostic spectrum, the analysis was primarily descriptive. No formal comparative statistical testing was performed.

### 2.11. Ethical Approval

The study was conducted in accordance with the principles of the Declaration of Helsinki and was approved by the local ethics committee of Ankara Etlik City Hospital with approval number AEŞH-BADEK2-2026-316. Because of the retrospective nature of the study and the use of anonymized archival material and clinical data, the requirement for written informed consent was waived by the ethics committee.

## 3. Results

### 3.1. Cohort Characteristics and Diagnostic Distribution

A total of 23 central nervous system tumors showing focal or predominant oligodendroglioma-like histomorphological features but not fulfilling the integrated diagnostic criteria for oligodendroglioma were included in the study. The median age at diagnosis was 35 years, ranging from 7 to 58 years. Fourteen patients were male and nine were female. The most frequent tumor locations were the frontal lobe in 10 cases, followed by the parietal lobe in six cases, temporal lobe in four cases, and intraventricular/lateral ventricular region in three cases.

The final integrated diagnoses included astrocytoma, IDH-mutant in nine cases, including CNS WHO grade 2 in four cases, grade 3 in one case, and grade 4 in four cases; dysembryoplastic neuroepithelial tumor in three; central neurocytoma in three; metastatic renal cell carcinoma in two; low-grade glioneuronal tumor in two; polymorphous low-grade neuroepithelial tumor of the young in one; glioblastoma, IDH-wildtype, CNS WHO grade 4, molecularly defined by *TERT* promoter mutation in one; supratentorial ependymoma in one; and pilocytic astrocytoma, *KIAA1549::BRAF* fusion-positive in one. When grouped according to broader diagnostic categories, glial tumors represented the largest group, followed by glioneuronal tumors, neuronal tumors, metastatic tumors, and ependymal tumors. Circumscribed gliomas were included within the broader glial tumor category. The overall clinicopathological and molecular characteristics of the cohort are summarized in Table 1, and detailed case-by-case clinicopathological, immunohistochemical, molecular, and follow-up data are provided in Supplementary Table S1.

### 3.2. Oligodendroglioma-like Histomorphological Features

All cases showed at least one histomorphological feature overlapping with oligodendroglioma. The most consistent finding was the presence of small monotonous round nuclei, observed in all 23 cases. Perinuclear halo or clearing was identified in 18 cases, chicken-wire-like capillary vasculature in 10 cases, calcification in nine cases, microcystic background in five cases, and a cortical infiltrative pattern in two cases.

**Figure 1:**
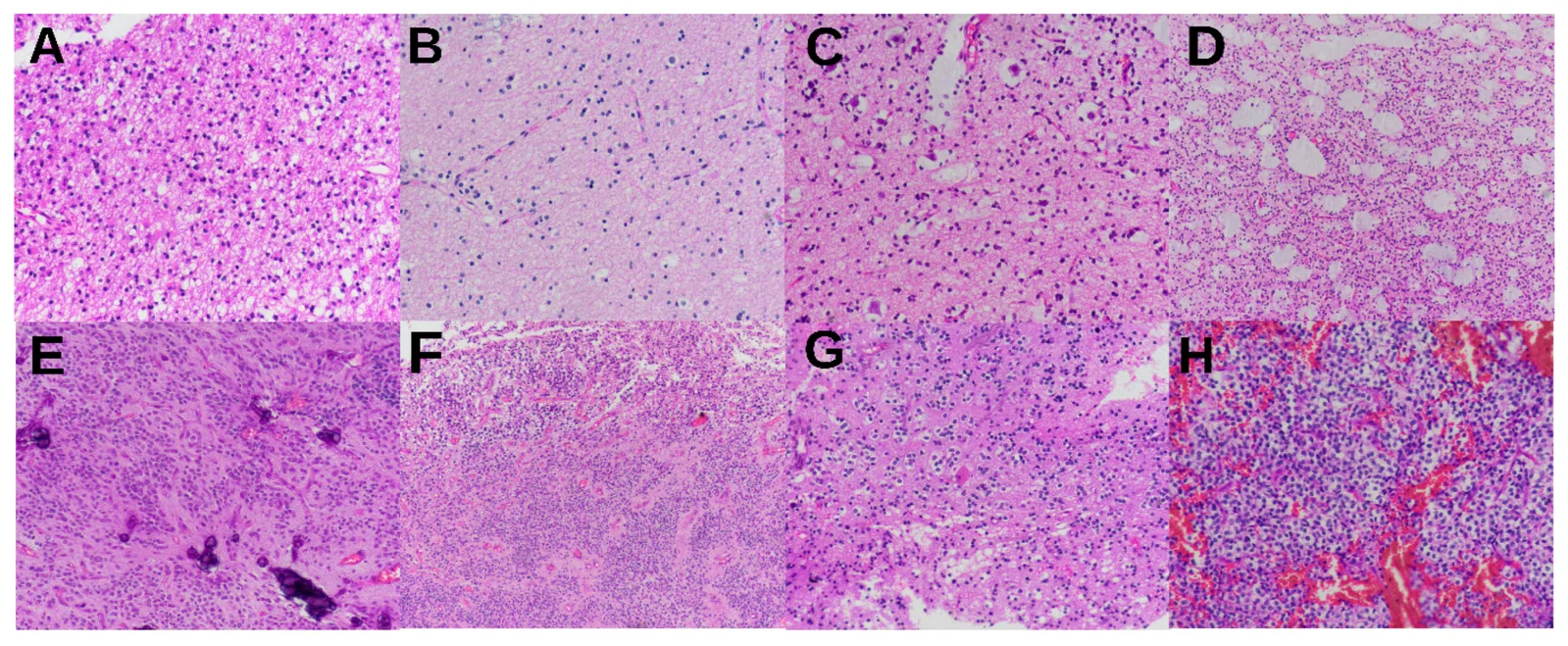
Representative histomorphological features of oligodendroglioma-like non-oligodendroglial tumors. (**A**) Diffuse astrocytoma, IDH-mutant, CNS WHO grade 2, showing relatively monotonous small round nuclei with focal perinuclear clearing/halo formation (H&E, 200×). (**B**) Dysembryoplastic neuroepithelial tumor (DNET), composed of small monotonous round cells with conspicuous perinuclear halos and a delicate chicken-wire capillary network (H&E, 100×). (**C**) Low-grade glioneuronal tumor, demonstrating atypical neurons together with small monotonous round cells, focal perinuclear halos, and delicate chicken-wire vasculature (H&E, 200×). (**D**) Low-grade glioneuronal tumor, showing a microcystic background with small monotonous round cells, focal perinuclear halos, and delicate chicken-wire capillaries (H&E, 100×). (**E**) Supratentorial ependymoma, composed of small round monotonous cells with focal calcifications and delicate chicken-wire-like vasculature (H&E, 200×). (**F**) Supratentorial ependymoma, showing oligodendroglioma-like areas with small round cells, some with perinuclear halos, admixed with more characteristic ependymal rosette/pseudorosette-like structures (H&E, 100×). (**G**) Polymorphous low-grade neuroepithelial tumor of the young (PLNTY), showing small monotonous round nuclei with conspicuous perinuclear halos (H&E, 200×). (**H**) Central neurocytoma, composed of small uniform round cells with perinuclear halos and delicate chicken-wire capillary vasculature (H&E, 200×).

Representative hematoxylin and eosin-stained sections are shown in Figure 1. Diffuse astrocytoma showed small round monotonous nuclei with focal perinuclear clearing. DNET and low-grade glioneuronal tumors demonstrated oligodendroglial-like cells, perinuclear halos, microcystic areas, delicate capillary networks, and admixed neuronal elements. The supratentorial ependymoma showed areas closely resembling oligodendroglioma, with small round cells, perinuclear clearing, calcification, and delicate capillaries, as well as more characteristic ependymal rosette/pseudorosette-like morphology. PLNTY showed small monotonous round nuclei with conspicuous perinuclear halos. Central neurocytoma demonstrated uniform small round cells, perinuclear halos, and delicate chicken-wire-like vasculature.

One pilocytic astrocytoma case showed focal oligodendroglioma-like morphology. Preoperative imaging described a subcortical mass lesion containing cystic and hemorrhagic components. Histologically, the oligodendroglioma-like component was focal and consisted of monotonous round nuclei, perinuclear halos, and calcification. The predominant morphology was classic biphasic pilocytic astrocytoma, with focal Rosenthal fibers and eosinophilic granular bodies. This case illustrates that oligodendroglioma-like morphology may also occur focally in circumscribed MAPK pathway–altered gliomas and may represent a diagnostic pitfall, particularly in limited biopsy samples.

### 3.3. Initial Microscopic Impressions and Diagnostic Reclassification

The initial microscopic impressions varied considerably across the cohort, reflecting the diagnostic uncertainty generated by oligodendroglioma-like morphology. The most common initial impressions were low-grade glial tumor in six cases (26.1%) and high-grade glial tumor in five cases (21.7%). Other initial impressions included DNET in three cases (13.0%), ependymoma in two cases (8.7%), astroblastoma in two cases (8.7%), central neurocytoma in one case (4.3%), dysplasia in one case (4.3%), oligodendroglioma in one case (4.3%), metastatic epithelial tumor in one case (4.3%), and pilocytic astrocytoma/low-grade glial tumor in one case (4.3%).

Comparison of initial microscopic impressions with final integrated diagnoses demonstrated that oligodendroglioma-like morphology did not lead to a single uniform diagnostic consideration, but rather generated a broad range of preliminary interpretations. Cases initially considered as low-grade glial tumors were finally classified as DNET, PLNTY, diffuse astrocytoma, or metastatic renal cell carcinoma. Cases initially considered as high-grade glial tumors were finally classified as diffuse astrocytoma or glioblastoma, IDH-wildtype, CNS WHO grade 4, molecularly defined by *TERT* promoter mutation. Two cases initially interpreted as ependymoma were finally classified as central neurocytoma, while two cases initially considered as astroblastoma were reclassified as supratentorial ependymoma and low-grade glioneuronal tumor, respectively. The single case initially considered as oligodendroglioma was finally classified as diffuse astrocytoma, IDH-mutant, after integrated molecular assessment. The distribution of initial microscopic impressions and their corresponding final integrated diagnoses is summarized in Table 2.

### 3.4. Immunohistochemical Findings

Immunohistochemistry contributed substantially to the final classification of the cases. In the diffuse astrocytoma group, IDH1 R132H positivity, *ATRX* loss, p53 nuclear positivity, and GFAP/Olig2 expression supported astrocytic lineage and helped distinguish these tumors from oligodendroglioma in the absence of 1p/19q codeletion. In the pilocytic astrocytoma case, GFAP, Olig2, and SOX10 positivity, together with a low Ki-67 proliferative index of approximately 4%, supported a circumscribed low-grade glioma. In glioneuronal tumors, neuronal differentiation was supported by synaptophysin and/or NeuN positivity, whereas CD34 expression was particularly helpful in the PLNTY case. Central neurocytomas showed neurocytic differentiation with synaptophysin and/or NeuN expression. The metastatic renal cell carcinoma cases were supported by renal epithelial markers, including PAX8, PAX2, CD10, vimentin, and epithelial marker expression according to the diagnostic work-up. The supratentorial ependymoma showed strong GFAP positivity, together with histomorphological features supporting ependymal differentiation.

**Figure 2:**
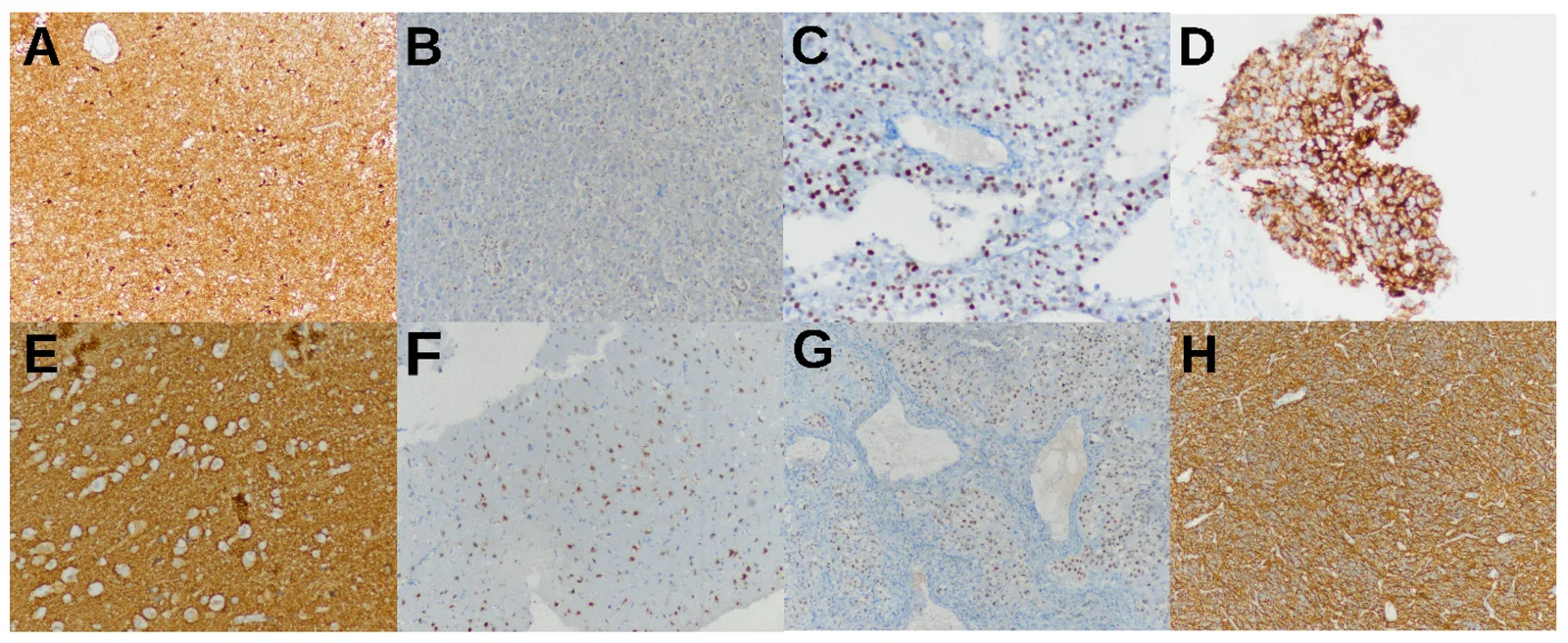
Representative immunohistochemical features of oligodendroglioma-like non-oligodendroglial tumors. (**A**) Diffuse astrocytoma, IDH-mutant, CNS WHO grade 2. Immunohistochemistry for IDH1 R132H shows cytoplasmic and nuclear positivity in tumor cells (100×). (**B**) Diffuse astrocytoma, IDH-mutant, CNS WHO grade 2. Immunohistochemistry for *ATRX* shows loss of nuclear expression in tumor cells (100×). (**C**) Diffuse astrocytoma, IDH-mutant, CNS WHO grade 2. Immunohistochemistry for p53 shows nuclear positivity in tumor cells (200×). (**D**) Polymorphous low-grade neuroepithelial tumor of the young (PLNTY). Immunohistochemistry for CD34 shows diffuse positivity in tumor cells (200×). (**E**) Low-grade glioneuronal tumor. Immunohistochemistry for synaptophysin shows positivity, supporting neuronal differentiation (200×). (**F**) Dysembryoplastic neuroepithelial tumor (DNET). Immunohistochemistry for NeuN highlights neuronal cells, supporting neuronal differentiation (100×). (**G**) Metastatic renal cell carcinoma. Immunohistochemistry for PAX8 shows nuclear positivity in tumor cells, supporting renal epithelial origin (100×). (**H**) Supratentorial ependymoma. Immunohistochemistry for GFAP shows strong diffuse positivity in tumor cells (100×).

Representative immunohistochemical findings are illustrated in Figure 2. IDH1 R132H positivity, *ATRX* loss, and p53 nuclear expression are shown in a diffuse astrocytoma. Diffuse CD34 positivity is demonstrated in PLNTY. Synaptophysin positivity in a low-grade glioneuronal tumor and NeuN-positive neuronal cells in DNET support neuronal/glioneuronal differentiation. Nuclear PAX8 positivity confirms renal epithelial differentiation in metastatic renal cell carcinoma, while strong GFAP positivity supports the diagnosis of supratentorial ependymoma.

### 3.5. Molecular and Cytogenetic Findings

Molecular and cytogenetic evaluation was available according to the original diagnostic work-up of each case. IDH mutation with absence of 1p/19q codeletion was identified in nine diffuse astrocytoma cases. The molecularly defined glioblastoma case showed *TERT* promoter mutation in the absence of IDH mutation and 1p/19q codeletion. A *ZFTA* alteration was detected in the supratentorial ependymoma case. *PRKCA*-related alteration/fusion was identified in two low-grade glioneuronal tumors. The pilocytic astrocytoma case harbored *KIAA1549::BRAF* fusion detected by next-generation sequencing; IDH mutation was not detected by NGS, and 1p/19q codeletion was not identified by fluorescence in situ hybridization. No pathogenic alteration was detected by NGS in nine cases.

No case in the cohort fulfilled the integrated molecular criteria for oligodendroglioma, IDH-mutant and 1p/19q-codeleted. Thus, molecular/cytogenetic assessment, particularly IDH status and 1p/19q evaluation, was essential in excluding true oligodendroglioma in tumors with overlapping morphology. In the pilocytic astrocytoma case, the presence of *KIAA1549::BRAF* fusion further supported a MAPK pathway–altered circumscribed glioma rather than oligodendroglioma. *CIC* and *FUBP1* testing was not systematically available; however, the absence of the required IDH-mutant and 1p/19q-codeleted profile was considered sufficient to exclude oligodendroglioma according to WHO CNS5 criteria. Methylation profiling was not systematically performed and therefore did not contribute to final classification in this cohort.

### 3.6. Diagnostic Contribution of Ancillary Testing

The contribution of ancillary testing was evaluated descriptively according to its role in diagnostic refinement or reclassification. IDH mutation analysis and 1p/19q status were central to excluding true oligodendroglioma and supporting astrocytic lineage in IDH-mutant diffuse gliomas. *ATRX* and p53 patterns further supported astrocytic differentiation in relevant cases. *TERT* promoter mutation supported the diagnosis of glioblastoma, IDH-wildtype, CNS WHO grade 4, molecularly defined by *TERT* promoter mutation, in one case. Synaptophysin and NeuN supported neuronal or glioneuronal differentiation in DNET, low-grade glioneuronal tumors, and central neurocytomas. CD34 positivity supported PLNTY in one case. GFAP expression and *ZFTA* alteration supported supratentorial ependymoma. PAX8, PAX2, CD10, vimentin, and epithelial marker expression supported metastatic renal cell carcinoma. *KIAA1549::BRAF* fusion supported pilocytic astrocytoma in the case with focal oligodendroglioma-like morphology. A case-group summary of the diagnostic evidence excluding oligodendroglioma and supporting the final integrated diagnoses is provided in Supplementary Table S2.

The diagnostic contribution of ancillary tests in the context of oligodendroglioma-like morphology is summarized in Table 3.

### 3.7. Follow-Up Findings

The median follow-up duration was 18 months, ranging from 6 to 48 months. Fourteen patients had no residual or recurrent disease during follow-up. Recurrent disease was documented in six cases, and three patients died during follow-up. The pilocytic astrocytoma case showed no postoperative residual tumor, and follow-up magnetic resonance imaging obtained two years after surgery demonstrated no evidence of recurrence. Follow-up status varied according to the final integrated diagnosis and biological behavior of the tumor, with adverse outcomes mainly observed among higher-grade glial tumors and metastatic disease. Follow-up data are summarized in Table 1 and detailed in Supplementary Table S1.

### 3.8. Practical Diagnostic Clues and Proposed Diagnostic Reappraisal Workflow

The practical differential diagnostic clues for oligodendroglioma-like central nervous system tumors are summarized in Table 4. In routine practice, diffuse astrocytoma should be considered when oligodendroglioma-like morphology is accompanied by IDH mutation, *ATRX* loss, p53 nuclear accumulation, and absence of 1p/19q codeletion. Pilocytic astrocytoma should be considered when oligodendroglioma-like areas are focal and coexist with classic biphasic architecture, Rosenthal fibers, eosinophilic granular bodies, low proliferative activity, and MAPK pathway alteration such as *KIAA1549::BRAF* fusion. DNET and other low-grade glioneuronal tumors should be considered in cortical lesions with microcystic architecture, neuronal elements, and synaptophysin/NeuN expression. PLNTY should be suspected in low-grade tumors showing oligodendroglioma-like morphology with diffuse or aberrant CD34 positivity. Central neurocytoma should be favored in intraventricular tumors with neurocytic immunophenotype. Supratentorial ependymoma should be considered when oligodendroglioma-like areas coexist with ependymal rosette/pseudorosette-like morphology and supportive ependymal/glial markers. Metastatic renal cell carcinoma should be excluded in clear-cell lesions with rich capillary vasculature, particularly when renal epithelial markers such as PAX8 are positive.

**Figure 3:**
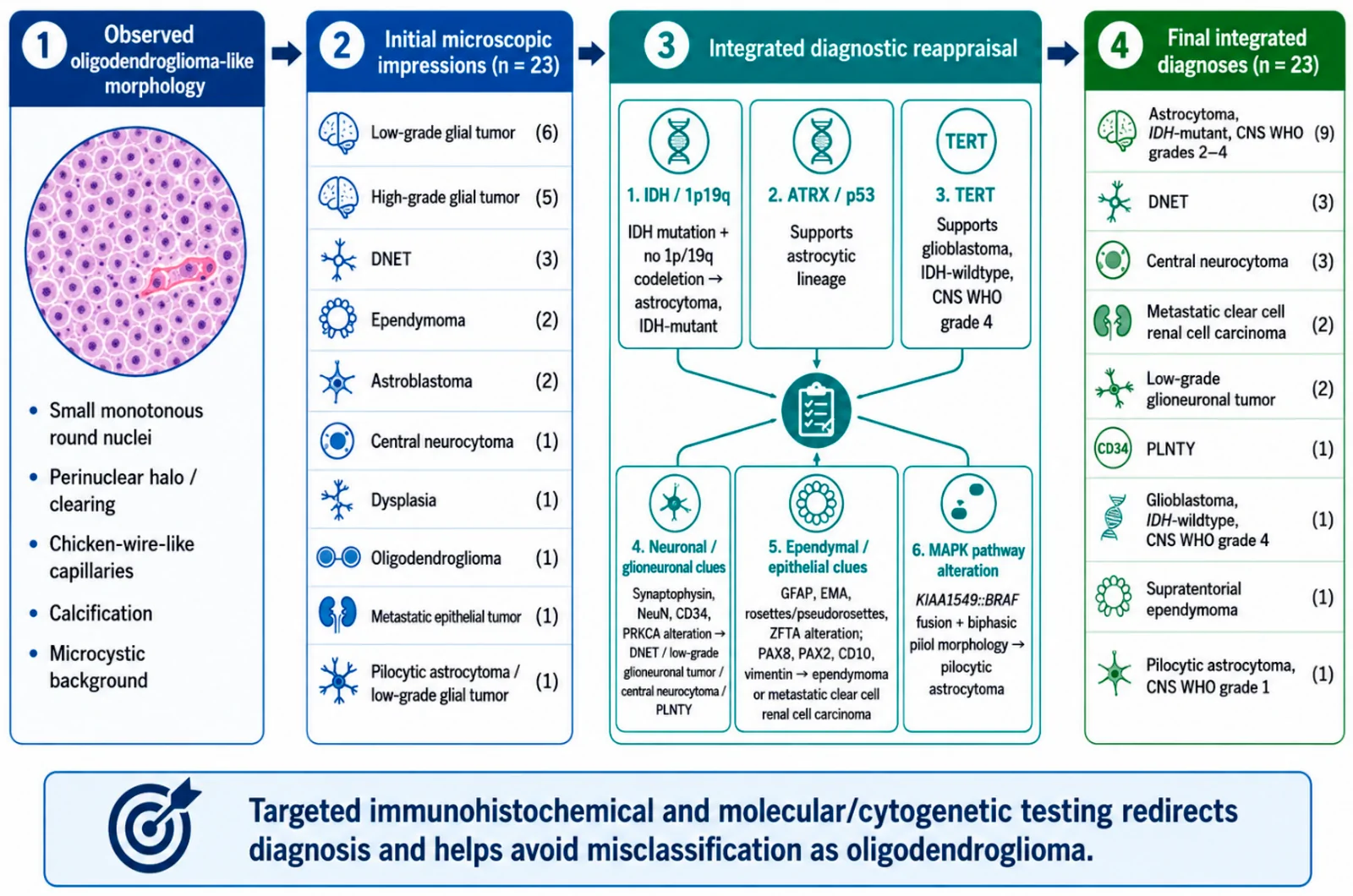
Diagnostic reappraisal workflow based on observed pitfalls. This figure summarizes the diagnostic reappraisal workflow for central nervous system tumors showing oligodendroglioma-like morphology in the present cohort. (**1**) The process begins with recognition of the main oligodendroglioma-like histomorphological features, including small monotonous round nuclei, perinuclear halo/clearing, delicate chicken-wire-like capillaries, calcification, and microcystic background. (**2**) These features generated a broad range of initial microscopic impressions, including low-grade glial tumor, high-grade glial tumor, dysembryoplastic neuroepithelial tumor (DNET), ependymoma, astroblastoma, central neurocytoma, dysplasia, oligodendroglioma, metastatic epithelial tumor, and pilocytic astrocytoma/low-grade glial tumor. (**3**) Integrated diagnostic reappraisal was then performed using targeted immunohistochemical and molecular/cytogenetic studies, including IDH/1p19q status, *ATRX*/p53, neuronal and glioneuronal markers, CD34, ependymal markers and *ZFTA* alteration, renal epithelial markers, and MAPK pathway alterations such as KIAA1549::BRAF fusion. (**4**) Final integrated diagnoses included astrocytoma, IDH-mutant, Grade 2–4; DNET; central neurocytoma; metastatic renal cell carcinoma; low-grade glioneuronal tumor; polymorphous low-grade neuroepithelial tumor of the young (PLNTY); glioblastoma, IDH-wildtype, CNS WHO grade 4; supratentorial ependymoma; and pilocytic astrocytoma. The workflow highlights that targeted ancillary testing redirects diagnosis and helps prevent misclassification of non-oligodendroglial tumors as oligodendroglioma.

Based on the observed diagnostic pitfalls and the reclassification patterns in this cohort, we propose a practical diagnostic reappraisal workflow for tumors showing oligodendroglioma-like morphology (Figure 3). Initial recognition of small monotonous round nuclei, perinuclear clearing, delicate branching capillaries, calcification, or microcystic change should prompt integrated evaluation rather than immediate classification as oligodendroglioma. The first diagnostic step should include assessment of IDH status and 1p/19q codeletion. In the absence of 1p/19q codeletion, *ATRX* and p53 patterns are useful for identifying astrocytic lineage. When circumscribed glioma is suspected, classic pilocytic astrocytoma morphology, low proliferative index, and MAPK pathway alterations such as *KIAA1549::BRAF* fusion should be evaluated. When glioneuronal differentiation is suspected, synaptophysin, NeuN, CD34, and relevant molecular alterations may support diagnoses such as DNET, PLNTY, or low-grade glioneuronal tumor. In intraventricular tumors, neurocytic markers help identify central neurocytoma. In tumors with clear-cell morphology or rich capillary vasculature, epithelial and renal markers should be included to exclude metastatic renal cell carcinoma. Ependymal morphology and markers, together with *ZFTA* alteration when present, support supratentorial ependymoma.

## 4. Discussion

In this single-center retrospective diagnostic reappraisal study, we analyzed 23 non-oligodendroglial central nervous system tumors that showed focal or predominant oligodendroglioma-like morphology but did not fulfill the integrated diagnostic criteria for oligodendroglioma. The cohort included a broad spectrum of final integrated diagnoses, comprising astrocytoma, IDH-mutant, CNS WHO grade 2–4; glioblastoma, IDH-wildtype, CNS WHO grade 4, molecularly defined by *TERT* promoter mutation; pilocytic astrocytoma with *KIAA1549::BRAF* fusion; dysembryoplastic neuroepithelial tumor (DNET); low-grade glioneuronal tumor; polymorphous low-grade neuroepithelial tumor of the young (PLNTY); central neurocytoma; supratentorial ependymoma; and metastatic renal cell carcinoma. The principal finding of this study is that oligodendroglioma-like morphology is not only a cross-entity histological pattern, but also a source of real-world diagnostic uncertainty during initial microscopic evaluation. This was reflected by the broad range of initial impressions in our cohort, including low-grade glial tumor, high-grade glial tumor, DNET, ependymoma, astroblastoma, oligodendroglioma, dysplasia, metastatic epithelial tumor, central neurocytoma, and pilocytic astrocytoma/low-grade glial tumor. The value of this study should therefore not be interpreted as demonstrating that molecular testing is superior to microscopy alone, which is already established in contemporary neuropathology practice, but as documenting how a single recurring morphologic pattern generates multiple diagnostic pathways in routine sign-out practice.

The diagnostic relevance of this observation lies in the contemporary definition of oligodendroglioma. Historically, oligodendroglioma was recognized by uniform round nuclei, perinuclear clearing, delicate branching capillaries, and calcification. However, in the molecular era, these features are insufficient for diagnosis. The 2021 World Health Organization classification defines oligodendroglioma by the combined presence of an IDH mutation and whole-arm 1p/19q codeletion [1]. Similarly, modern glioma guidelines and cIMPACT-NOW recommendations emphasize integrated histomolecular interpretation, particularly when histological features are limited, overlapping, or discordant with molecular findings [2,3]. Our findings support this principle: no case in the cohort fulfilled the integrated molecular criteria for oligodendroglioma, despite the presence of oligodendroglioma-like features in all cases. In this context, CIC and FUBP1 alterations should be interpreted as frequent and diagnostically supportive molecular events in oligodendroglioma, but not as defining criteria in the absence of the essential IDH-mutant and whole-arm 1p/19q-codeleted profile. Accordingly, the lack of systematic CIC/FUBP1 testing in the present cohort does not invalidate the exclusion of oligodendroglioma in tumors that lacked the required WHO CNS5-defining molecular profile.

The most frequent final diagnostic category in our series was diffuse astrocytoma, IDH-mutant. Recent updates on IDH-mutant glioma diagnosis further emphasize the integrated interpretation of IDH status, 1p/19q codeletion, *ATRX* expression, p53 expression, and *TERT* promoter status in distinguishing astrocytoma from oligodendroglioma [6]. This is diagnostically important because IDH-mutant diffuse astrocytomas may show areas with small round cells, perinuclear clearing, delicate capillaries, and calcification, thereby resembling oligodendroglioma on hematoxylin and eosin sections. In our cohort, the combination of IDH mutation, absence of 1p/19q codeletion, and supportive immunohistochemical findings such as *ATRX* loss and p53 nuclear expression redirected classification toward astrocytic lineage. This distinction is clinically relevant because IDH-mutant astrocytoma and oligodendroglioma differ in prognosis, expected treatment response, grading approach, and follow-up strategy [1,2,23]. Therefore, when oligodendroglioma-like morphology is encountered in a diffuse glioma, IDH testing and 1p/19q assessment should be regarded as essential rather than optional diagnostic steps.

The inclusion of one glioblastoma, IDH-wildtype, CNS WHO grade 4, molecularly defined by *TERT* promoter mutation further illustrates the impact of molecular testing in this morphological setting. cIMPACT-NOW update 3 established that IDH-wildtype diffuse astrocytic gliomas harboring molecular features such as *TERT* promoter mutation, EGFR amplification, or combined whole chromosome 7 gain and chromosome 10 loss may correspond biologically to glioblastoma, even when classic histological features are limited or heterogeneous [3]. In our cohort, the glioblastoma case showed oligodendroglioma-like areas with small round cells, perinuclear clearing, calcification, and delicate vasculature, but the absence of IDH mutation and 1p/19q codeletion together with *TERT* promoter mutation supported the final diagnosis of glioblastoma, IDH-wildtype, CNS WHO grade 4, molecularly defined by *TERT* promoter mutation. This finding highlights that oligodendroglioma-like morphology can occur not only in low-grade mimics, but also in biologically high-grade diffuse gliomas.

The pilocytic astrocytoma case expands the diagnostic spectrum by showing that focal oligodendroglioma-like morphology may also occur in circumscribed MAPK pathway–altered gliomas. In this case, the oligodendroglioma-like component consisted of monotonous round nuclei, perinuclear halos, and calcification, whereas the predominant tumor architecture showed classic biphasic pilocytic astrocytoma morphology with Rosenthal fibers and eosinophilic granular bodies. GFAP, Olig2, and SOX10 positivity, low Ki-67 proliferative activity, absence of IDH mutation and 1p/19q codeletion, and the presence of *KIAA1549::BRAF* fusion supported the final diagnosis. *KIAA1549::BRAF* fusion is a well-established molecular alteration in pilocytic astrocytoma and other MAPK pathway–altered low-grade gliomas [11,12,13,14,15]. Although the diagnosis was straightforward in the resection specimen, this case underscores a practical pitfall: in limited biopsies, focal oligodendroglioma-like areas may be overinterpreted if classic piloid features are underrepresented.

Low-grade glioneuronal and epilepsy-associated tumors represented another important diagnostic group. DNET has long been recognized as a cortically based, epilepsy-associated tumor that may contain oligodendroglial-like cells, floating neurons, and microcystic architecture [7,8]. In our series, DNET and low-grade glioneuronal tumors showed several overlapping features with oligodendroglioma, including small monotonous round nuclei, perinuclear halos, microcystic background, and delicate capillary networks. However, the presence of neuronal elements and immunohistochemical support by NeuN and/or synaptophysin redirected diagnosis toward glioneuronal differentiation. In selected low-grade glioneuronal tumors, *PRKCA*-related alteration or fusion further supported a non-oligodendroglial diagnosis. These findings indicate that neuronal and glioneuronal markers should be actively incorporated into the work-up of cortically based oligodendroglioma-like lesions, especially in young patients or epilepsy-associated presentations.

PLNTY is another diagnostically relevant mimic in this setting. Since its initial description, PLNTY has been characterized by oligodendroglioma-like components, frequent calcification, aberrant CD34 expression, and MAPK pathway alterations [9]. In our PLNTY case, the tumor showed small monotonous round nuclei, perinuclear halos, and calcification, closely resembling oligodendroglioma morphologically. However, diffuse CD34 positivity, absence of 1p/19q codeletion, and the overall low-grade neuroepithelial tumor context supported PLNTY. Imaging-based studies have also emphasized calcification as a frequent radiological clue in PLNTY, further contributing to potential overlap with oligodendroglioma [10]. Therefore, CD34 staining and MAPK pathway evaluation, when available, may be useful in low-grade cortical tumors with oligodendroglioma-like features.

Central neurocytoma also emerged as a meaningful mimic in our cohort. The uniform small round cell morphology, perinuclear halo-like clearing, and delicate capillary network of central neurocytoma may closely resemble oligodendroglioma, particularly when the intraventricular location is not fully appreciated or when biopsy material is limited. In our cases, intraventricular/lateral ventricular location and neurocytic immunophenotype supported by synaptophysin and/or NeuN were decisive for diagnosis. Previous reviews have similarly emphasized the role of neurocytic differentiation in distinguishing central neurocytoma from oligodendroglioma and other clear-cell primary brain tumors [16]. This highlights the importance of integrating anatomical location into histological interpretation.

Supratentorial ependymoma was another diagnostically informative entity. Clear cell ependymoma is a recognized mimic of oligodendroglioma because of its small round cells, clear-cell appearance, perinuclear halo-like change, calcification, and delicate vasculature [18,19]. In our case, oligodendroglioma-like areas coexisted with more characteristic ependymal rosette/pseudorosette-like morphology. Strong GFAP expression and *ZFTA* alteration supported supratentorial ependymoma. This case illustrates that architectural clues beyond the oligodendroglioma-like component must be carefully sought, because ependymal differentiation may be focal and easily overlooked. Although ependymal tumor grading has limitations and reproducibility issues, recognition of ependymal architecture and molecular subgroup-related alterations remains important for integrated diagnosis [17].

The metastatic renal cell carcinoma cases further demonstrate that oligodendroglioma-like morphology is not restricted to primary neuroepithelial tumors. Clear cell renal cell carcinoma metastasis may mimic oligodendroglioma through clear cytoplasm, perinuclear clearing, and a rich delicate vascular network. This overlap may be particularly problematic in small biopsies or in patients without a known renal primary at the time of neuropathological evaluation. In our cases, PAX8, PAX2, CD10, vimentin, and epithelial marker expression supported metastatic renal cell carcinoma and helped exclude a primary glial neoplasm. Therefore, renal epithelial markers should be considered when clear-cell morphology and rich capillary vasculature are present, especially if GFAP and Olig2 are absent or the clinical/radiological context is atypical for a diffuse glioma.

Neuroimaging also remains an important component of the differential diagnosis in tumors with oligodendroglioma-like morphology. In the present cohort, tumor location and radiological context were particularly helpful for selected entities, such as intraventricular localization in central neurocytoma, cortical-based lesions in DNET and other low-grade glioneuronal tumors, and clear-cell metastatic tumors requiring systemic correlation. Recent MRI-based studies have also investigated imaging parameters that may help differentiate IDH-mutant astrocytoma from 1p/19q-codeleted oligodendroglioma, further supporting the value of clinicoradiological correlation as part of integrated diagnosis [24].

A key contribution of the present study is the comparison of initial microscopic impressions with final integrated diagnoses. The most common initial impressions were low-grade glial tumor and high-grade glial tumor, but several cases were initially considered as DNET, ependymoma, astroblastoma, oligodendroglioma, dysplasia, metastatic epithelial tumor, central neurocytoma, or pilocytic astrocytoma/low-grade glial tumor. This distribution shows that oligodendroglioma-like morphology does not generate a single diagnostic bias, but rather produces a broad field of preliminary interpretations. In this context, the value of ancillary testing lies not only in confirming a suspected diagnosis, but also in redirecting broad or misleading initial impressions toward a precise integrated diagnosis. Thus, the practical added value of the present series lies in documenting the diagnostic pathways generated by oligodendroglioma-like morphology and summarizing the ancillary clues that may help prioritize the differential diagnosis in limited or morphologically overlapping samples.

The diagnostic contribution of ancillary tests was therefore central to this study. IDH mutation and 1p/19q status excluded true oligodendroglioma and supported astrocytic lineage in IDH-mutant diffuse gliomas. *ATRX* and p53 supported astrocytic differentiation in selected cases. *TERT* promoter mutation supported glioblastoma, IDH-wildtype, CNS WHO grade 4, molecularly defined by *TERT* promoter mutation. Synaptophysin, NeuN, and TTF1 supported neuronal or glioneuronal differentiation in DNET, central neurocytoma, and selected low-grade glioneuronal tumors. CD34 positivity supported PLNTY. GFAP and *ZFTA* alteration supported supratentorial ependymoma. PAX8, PAX2, CD10, vimentin, and epithelial marker expression supported metastatic renal cell carcinoma. *KIAA1549::BRAF* fusion supported pilocytic astrocytoma. This ancillary test-based reappraisal provides a practical framework for avoiding overdiagnosis of oligodendroglioma in tumors with overlapping morphology. This is consistent with the contemporary molecular neuropathology approach, in which targeted sequencing, copy-number assessment, fusion testing, and DNA methylation profiling are increasingly used as complementary tools for resolving diagnostically challenging CNS tumors [25]. The final diagnoses were therefore not based solely on the absence of oligodendroglioma-defining markers, but on positive alternative diagnostic evidence, including astrocytic immunophenotype and IDH-mutant/non-codeleted status, neuronal or glioneuronal differentiation, CD34 expression, renal epithelial marker expression, *ZFTA* alteration, *PRKCA*-related alteration/fusion, *TERT* promoter mutation, or *KIAA1549::BRAF* fusion, depending on the case.

Based on these findings, we propose a diagnostic reappraisal workflow rather than a purely theoretical differential diagnostic algorithm. The workflow begins with recognition of oligodendroglioma-like morphology on routine sections, followed by documentation of the initial microscopic impression and targeted integrated reassessment. The first essential step is evaluation of IDH status and 1p/19q codeletion. IDH-mutant tumors lacking 1p/19q codeletion should be assessed for astrocytic lineage using *ATRX* and p53. IDH-wildtype or molecularly non-diagnostic tumors require broader interpretation according to architecture, location, lineage-specific immunohistochemistry, and targeted molecular alterations. Cortical microcystic tumors with neuronal elements should raise DNET or other low-grade glioneuronal tumors; CD34-positive low-grade tumors should suggest PLNTY; intraventricular tumors with neurocytic markers should favor central neurocytoma; tumors with rosettes/pseudorosettes and *ZFTA* alteration should suggest supratentorial ependymoma; clear-cell lesions with renal epithelial markers should prompt evaluation for metastatic renal cell carcinoma; and tumors with biphasic piloid morphology and *KIAA1549::BRAF* fusion should support pilocytic astrocytoma.

This study has several limitations. First, it is retrospective and single-center in design, with a relatively small cohort. Second, the cohort is diagnostically heterogeneous, which reflects real-world neuropathology practice but limits formal statistical comparison between individual entities. Third, molecular testing was performed according to the original diagnostic work-up and was not uniform across all cases. CIC and FUBP1 testing and genome-wide DNA methylation profiling were not systematically available across the cohort. We acknowledge that this limits the molecular resolution of selected diagnostically challenging tumors, particularly within the glioneuronal, circumscribed glioma, and pediatric-type low-grade neuroepithelial tumor spectrum. Therefore, the proposed workflow should not be interpreted as a replacement for methylation profiling when such testing is clinically indicated or available [25]. Rather, it reflects a practical reappraisal approach based on routinely available histopathological, immunohistochemical, cytogenetic, and targeted molecular data. Fourth, the definition of oligodendroglioma-like morphology was based on histopathological review and may be subject to interobserver variability. Finally, this study was not designed as a formal diagnostic performance analysis; sensitivity, specificity, and predictive values of the proposed workflow were not calculated. Nevertheless, by documenting initial microscopic impressions, final integrated diagnoses, and the contribution of ancillary testing, this study provides practical diagnostic information applicable to routine neuropathology practice.

## 5. Conclusions

Oligodendroglioma-like morphology should be regarded as a diagnostic pattern rather than a tumor-specific feature. In this cohort, it generated a broad range of initial microscopic impressions and was ultimately associated with diverse final integrated diagnoses, including diffuse glioma, circumscribed glioma, glioneuronal, neuronal, ependymal, and metastatic tumors. Although integrated histomolecular diagnosis is already the standard for CNS tumors, this case-based reappraisal illustrates the practical diagnostic trajectories created by oligodendroglioma-like morphology and highlights entity-specific ancillary clues that may support diagnostic refinement in routine neuropathology practice. In cases with persistent diagnostic ambiguity, especially within the glioneuronal or circumscribed glioma spectrum, DNA methylation profiling may provide additional diagnostic resolution when available.

## Figures and Tables

**Table 1:** Summary of clinicopathological and molecular characteristics of 23 oligodendroglioma-like non-oligodendroglial CNS tumors.

Parameter	*n* (%) or Median (Range)
**Total number of cases**	23
**Age, years**	35 (7–58)
**Sex**	
Male	14 (60.9)
Female	9 (39.1)
**Tumor location**	
Frontal lobe	10 (43.5)
Parietal lobe	6 (26.1)
Temporal lobe	4 (17.4)
Intraventricular/lateral ventricular region	3 (13.0)
**Main diagnostic category**	
Glial tumors	11 (47.8)
Glioneuronal tumors	6 (26.1)
Neuronal tumors	3 (13.0)
Metastatic tumors	2 (8.7)
Ependymal tumors	1 (4.3)
**Final integrated diagnosis**	
Astrocytoma, IDH-mutant, CNS WHO grade 2/3/4	9 (39.1)
Dysembryoplastic neuroepithelial tumor, CNS WHO grade 1	3 (13.0)
Central neurocytoma, CNS WHO grade 2	3 (13.0)
Metastatic renal cell carcinoma	2 (8.7)
Low-grade glioneuronal tumor	2 (8.7)
Polymorphous low-grade neuroepithelial tumor of the young	1 (4.3)
Glioblastoma, IDH-wildtype, CNS WHO grade 4	1 (4.3)
Supratentorial ependymoma	1 (4.3)
Pilocytic astrocytoma, CNS WHO grade 1, *KIAA1549::BRAF* fusion-positive	1 (4.3)
**Oligodendroglioma-like histomorphological features**	
Small monotonous round nuclei	23 (100)
Perinuclear halo/clearing	18 (78.3)
Chicken-wire-like capillary vasculature	10 (43.5)
Calcification	9 (39.1)
Microcystic background	5 (21.7)
Cortical infiltrative pattern	2 (8.7)
**Molecular/cytogenetic findings**	
IDH mutation with absence of 1p/19q codeletion	9 (39.1)
*TERT* promoter mutation with absence of IDH mutation and 1p/19q codeletion	1 (4.3)
*PRKCA* alteration/fusion	2 (8.7)
*ZFTA* alteration	1 (4.3)
KIAA1549::BRAF fusion	1 (4.3)
No pathogenic alteration detected by NGS	9 (39.1)
**Ki-67 proliferative index, %**	5 (1–20)
**Follow-up duration, months**	18 (6–48)
**Clinical follow-up status**	
No residual or recurrent disease	14 (60.9)
Recurrent disease	6 (26.1)
Death during follow-up	3 (13.0)

**Note:** Percentages are rounded to one decimal place and may not total exactly 100%. Abbreviations: CNS, central nervous system; IDH, isocitrate dehydrogenase; NGS, next-generation sequencing.

**Table 2:** Initial microscopic impressions and final integrated diagnoses.

Initial Microscopic Impression	Cases, *n* (%)	Final Integrated Diagnoses
Low-grade glial tumor	6 (26.1)	DNET (*n* = 1); PLNTY (*n* = 1); diffuse astrocytoma, IDH-mutant (*n* = 3); metastatic renal cell carcinoma (*n* = 1)
High-grade glial tumor	5 (21.7)	Diffuse astrocytoma, IDH-mutant (*n* = 4); glioblastoma, IDH-wildtype, CNS WHO grade 4 (*n* = 1)
Dysembryoplastic neuroepithelial tumor	3 (13.0)	Dysembryoplastic neuroepithelial tumor (*n* = 2); diffuse astrocytoma, IDH-mutant (*n* = 1)
Ependymoma	2 (8.7)	Central neurocytoma (*n* = 2)
Astroblastoma	2 (8.7)	Supratentorial ependymoma (*n* = 1); low-grade glioneuronal tumor (*n* = 1)
Central neurocytoma	1 (4.3)	Central neurocytoma (*n* = 1)
Dysplasia	1 (4.3)	Low-grade glioneuronal tumor (*n* = 1)
Oligodendroglioma	1 (4.3)	Diffuse astrocytoma, IDH-mutant (*n* = 1)
Metastatic epithelial tumor	1 (4.3)	Metastatic renal cell carcinoma (*n* = 1)
Pilocytic astrocytoma/low-grade glial tumor	1 (4.3)	Pilocytic astrocytoma, KIAA1549::BRAF fusion-positive (*n* = 1)
Total	23 (100)	

**Note:** Percentages are rounded to one decimal place and may not total exactly 100%. Abbreviations: IDH, isocitrate dehydrogenase; PLNTY, polymorphous low-grade neuroepithelial tumor of the young.

**Table 3:** Diagnostic contribution of ancillary tests in oligodendroglioma-like CNS tumors.

Ancillary Test/Marker Group	Relevant Cases, *n*	Diagnostic Contribution
*IDH* mutation analysis and 1p/19q codeletion status	9	Excluded oligodendroglioma and supported astrocytoma, IDH-mutant.
*TERT* promoter mutation with absence of IDH mutation and 1p/19q codeletion	1	Supported glioblastoma, IDH-wildtype, CNS WHO grade 4.
*ATRX* and p53 immunohistochemistry	Selected diffuse glioma cases	Supported astrocytic lineage.
NeuN, synaptophysin and TTF1	7	Supported neuronal or glioneuronal differentiation.
CD34 immunohistochemistry	1	Supported PLNTY.
*PRKCA*-related alteration/fusion	2	Supported low-grade glioneuronal tumor.
GFAP, Olig2 and SOX10 with KIAA1549::BRAF fusion	1	Supported pilocytic astrocytoma, CNS WHO grade 1.
GFAP and *ZFTA* alteration	1	Supported supratentorial ependymoma.
PAX8, PAX2, CD10 and vimentin	2	Supported metastatic clear cell renal cell carcinoma.
No pathogenic alteration detected by NGS	9	Helped exclude tested tumor-defining molecular alterations and contributed to integrated interpretation in selected non-oligodendroglial tumors.

Abbreviations: CNS, central nervous system; GFAP, glial fibrillary acidic protein; IDH, isocitrate dehydrogenase; NGS, next-generation sequencing; Olig2, oligodendrocyte transcription factor 2; PLNTY, polymorphous low-grade neuroepithelial tumor of the young; SOX10, SRY-box transcription factor 10; TTF1, thyroid transcription factor 1.

**Table 4:** Practical differential diagnostic clues in oligodendroglioma-like CNS tumors.

Final Diagnosis	Oligodendroglioma-like Histomorphological Clues	Key Immunohistochemical Clues	Key Molecular/Diagnostic Clues
Astrocytoma, IDH-mutant, Grade 2–4	Small monotonous round nuclei, perinuclear halo/clearing, delicate capillary vasculature; may closely mimic oligodendroglioma, especially in limited or heterogeneous samples	IDH1 R132H positivity, *ATRX* loss when present, p53 nuclear positivity, GFAP and Olig2 positivity	IDH mutation with absence of 1p/19q codeletion supports astrocytic lineage rather than oligodendroglioma
Glioblastoma, IDH-wildtype, CNS WHO grade 4	Oligodendroglioma-like areas with small round cells, perinuclear clearing, calcification, and delicate vasculature; high-grade features may be focal or underrepresented	GFAP/Olig2 expression may be present; p53 and *ATRX* patterns may vary; Ki-67 may be increased compared with low-grade mimics	IDH-wildtype status with molecular glioblastoma-defining alteration, such as *TERT* promoter mutation, supports diagnosis despite oligodendroglioma-like morphology
Pilocytic astrocytoma, KIAA1549::BRAF fusion-positive	Focal oligodendroglioma-like areas with monotonous round nuclei, perinuclear halos, and calcification; predominant classic biphasic architecture, Rosenthal fibers, and eosinophilic granular bodies favor pilocytic astrocytoma	GFAP, Olig2, and SOX10 positivity; low Ki-67 proliferative index	IDH-wildtype status, absence of 1p/19q codeletion, and KIAA1549::BRAF fusion support a circumscribed MAPK pathway–altered glioma rather than oligodendroglioma
Dysembryoplastic neuroepithelial tumor	Oligodendroglial-like cells, perinuclear halos, microcystic background, delicate chicken-wire-like capillary network	NeuN-positive neuronal cells; synaptophysin may highlight neuronal/glioneuronal component; variable GFAP/Olig2 expression	Cortical-based lesion, frequent epilepsy-associated setting, low-grade glioneuronal architecture, and absence of integrated molecular features of oligodendroglioma support DNET
Low-grade glioneuronal tumor	Small monotonous round cells, perinuclear halos, microcystic background, chicken-wire-like vasculature, admixed atypical neuronal elements	Synaptophysin positivity supports neuronal differentiation; GFAP/Olig2 may highlight glial component	Glioneuronal differentiation and, when present, *PRKCA*-related alteration/fusion support a non-oligodendroglial diagnosis
Polymorphous low-grade neuroepithelial tumor of the young	Oligodendroglioma-like cells, perinuclear halos, calcification, and low-grade morphology	Diffuse or aberrant CD34 positivity; GFAP/Olig2 may be positive; low Ki-67 index	IDH-wildtype status and absence of 1p/19q codeletion; MAPK pathway alterations, when present, support PLNTY
Central neurocytoma	Uniform small round cells, perinuclear halos, clear-cell-like appearance, delicate chicken-wire-like capillary network	Synaptophysin and NeuN positivity support neurocytic differentiation; GFAP is usually absent or limited to background/reactive glial cells	Intraventricular/lateral ventricular location and neurocytic immunophenotype help distinguish it from oligodendroglioma
Supratentorial ependymoma	Small round monotonous cells, clear-cell/perinuclear halo-like appearance, calcification, delicate capillary network; may show oligodendroglioma-like areas	GFAP positivity; EMA dot-like/ring-like staining if present; ependymal rosette or pseudorosette morphology supports diagnosis	*ZFTA* alteration/fusion, when detected, supports supratentorial ependymoma; ependymal architecture helps exclude oligodendroglioma
Metastatic renal cell carcinoma	Clear-cell morphology, perinuclear clearing, and rich delicate capillary network may mimic oligodendroglioma, especially in limited biopsy material	PAX8, PAX2, pan-cytokeratin, CD10, and vimentin positivity support renal epithelial origin; GFAP/Olig2 negativity favors metastasis	Clinical/radiological correlation and renal primary tumor evaluation are essential; epithelial and renal markers exclude primary glial tumor

Abbreviations: CNS, central nervous system; DNET, dysembryoplastic neuroepithelial tumor; GFAP, glial fibrillary acidic protein; IDH, isocitrate dehydrogenase; Olig2, oligodendrocyte transcription factor 2; PLNTY, polymorphous low-grade neuroepithelial tumor of the young; SOX10, SRY-box transcription factor 10.

## Data Availability

The original contributions presented in this study are included in the article/Supplementary Material. Further inquiries can be directed to the corresponding author.

## Supplementary Materials

The following supporting information can be downloaded at: https://www.mdpi.com/article/10.3390/diagnostics16172723/s1, Table S1: Detailed case-by-case clinicopathological, immunohistochemical, molecular, and follow-up data of 23 non-oligodendroglial CNS tumors with oligodendroglioma-like morphology. Table S2. Diagnostic evidence excluding oligodendroglioma and supporting final integrated diagnoses.
