# Supplementary material for: Diagnostic Pitfalls of Oligodendroglioma-like Morphology: Integrated Reappraisal of 23 Non-Oligodendroglial Central Nervous System Tumors
Source: Diagnostics (Basel). 2026 Aug 26;16(17):2723. doi: 10.3390/diagnostics16172723 (PMC13564585; doi:10.3390/diagnostics16172723)
Supplement: Supplementary file 1 [file diagnostics-16-02723-s001.zip › Supplementary Table S1.pdf]

Supplementary Table S1. Detailed case-by-case clinicopathological, immunohistochemical, molecular, and follow-up data of 23 non-oligodendroglial CNS tumors with oligodendroglioma-like morphology

Case	Age / sex	Location	Radiological summary	Initial microscopic impression	Oligodendroglioma-like features	Positive IHC markers	Molecular / FISH / NGS findings	Final integrated diagnosis	Ki-67	Follow-up
1	15 / M	Frontal lobe	Left frontal hypodense mass measuring 27 × 22 mm with cortical thinning	Low-grade glial tumor	Small monotonous round nuclei, perinuclear halos, microcystic background	Olig2, NeuN	No pathogenic alteration detected by NGS; no 1p/19q codeletion by FISH	Dysembryoplastic neuroepithelial tumor	4%	No residual/recurrent disease at postoperative year 3
2	47 / F	Parietal lobe	Right parietal cystic mass with linear calcifications and multiple septa	Low-grade glial tumor	Small monotonous round nuclei, perinuclear halos, microcystic background, chicken-wire capillaries, calcification, cortical infiltrative pattern	GFAP, Olig2, CD34	No pathogenic alteration detected by NGS; no 1p/19q codeletion by FISH	PLNTY	1%	No residual/recurrent disease at postoperative month 6
3	35 / M	Frontal lobe	Right frontal cystic-solid mass with mild diffusion restriction, edema, and midline shift	High-grade glial tumor	Small monotonous round nuclei, chicken-wire capillaries, microcystic background, initial oligodendroglioma consideration	GFAP, Olig2, IDH1 R132H	IDH mutation detected by NGS; no 1p/19q codeletion by FISH	Diffuse astrocytoma, IDH-mutant, CNS WHO grade 2	7%	Recurrence at postoperative month 18
4	48 / M	Temporal lobe	Left temporal cortical-subcortical non-enhancing lesion with cystic-necrotic/hemorrhagic areas	Low-grade glial tumor	Small monotonous round nuclei, initial oligodendroglioma consideration, calcification	GFAP, Olig2, IDH1 R132H	IDH mutation detected by NGS; no 1p/19q codeletion by FISH	Diffuse astrocytoma, IDH-mutant, CNS WHO grade 2	5%	No residual/recurrent disease at postoperative year 4
5	31 / M	Parietal lobe	Left posterior cortical-subcortical non-enhancing lesion; radiological differential included FCD, low-grade glioma, and DNET	DNET	Small monotonous round nuclei, perinuclear halos, chicken-wire capillaries, microcystic background	GFAP, Olig2, IDH1 R132H	IDH mutation detected by NGS; no 1p/19q codeletion by FISH	Diffuse astrocytoma, IDH-mutant, CNS WHO grade 3	15 %	Recurrence at postoperative month 9
6	36 / M	Frontal lobe	Left frontotemporal heterogeneous hypodense mass compressing the anterior horn of the lateral ventricle	Low-grade glial tumor	Small monotonous round nuclei, perinuclear halos, calcification	GFAP, Olig2	IDH mutation detected by NGS; no 1p/19q codeletion by FISH	Diffuse astrocytoma, IDH-mutant, CNS WHO grade 3	12 %	No residual/recurrent disease at postoperative year 3
7	53 / M	Frontal lobe	Right frontal T1-hypointense lesion with calcification and no prominent contrast enhancement	Low-grade glial tumor	Small monotonous round nuclei, perinuclear halos, chicken-wire capillaries, microcystic background, calcification,	GFAP, Olig2, IDH1 R132H, p53, ATRX	IDH mutation detected by NGS; no 1p/19q codeletion by FISH	Diffuse astrocytoma, IDH-mutant, CNS WHO grade 3	15 %	Recurrence at postoperative year 2

Case	Age / sex	Location	Radiological summary	Initial microscopic impression	Oligodendroglioma-like features	Positive IHC markers	Molecular / FISH / NGS findings	Final integrated diagnosis	Ki-67	Follow-up
					initial oligodendroglioma consideration					
8	41 / M	Parietal lobe	Left parietal calcified hyperdense lesion with hemorrhagic foci and surrounding edema	High-grade glial tumor	Small monotonous round nuclei, perinuclear halos, calcification, chicken-wire capillaries	GFAP, Olig2, IDH1 R132H	IDH mutation detected by NGS; no 1p/19q codeletion by FISH	Diffuse astrocytoma, IDH-mutant, CNS WHO grade 4	20 %	Recurrence at postoperative year 1
9	58 / M	Parietal lobe	Right parietal lobulated mass with hemorrhagic-necrotic areas	Low-grade glial tumor	Small monotonous round nuclei, perinuclear halos	PAX8, vimentin, PAX2	No pathogenic alteration detected by NGS	Metastatic renal cell carcinoma	15 %	Died at postoperative month 6
10	51 / M	Temporal lobe	Left temporal mass with extensive edema, hemorrhagic components, peripheral/nodular enhancement, and midline shift	High-grade glial tumor	Small monotonous round nuclei, calcification, microcystic background	GFAP, Olig2, IDH1 R132H	IDH mutation detected by NGS; no 1p/19q codeletion by FISH	Diffuse astrocytoma, IDH-mutant, CNS WHO grade 4	20 %	Died at postoperative year 1
11	44 / M	Temporal lobe	Right temporoparietal mass with focal nodular enhancement	High-grade glial tumor	Small monotonous round nuclei, chicken-wire capillaries	GFAP, Olig2, IDH1 R132H	IDH mutation detected by NGS; no 1p/19q codeletion by FISH	Diffuse astrocytoma, IDH-mutant, CNS WHO grade 2	2%	No residual/recurrent disease at postoperative year 3
12	15 / F	Frontal lobe	Left frontal cortical lesion with foamy/multicystic contour, radiologically favored DNET	DNET	Small monotonous round nuclei, perinuclear halos	Olig2, NeuN, S100	No pathogenic alteration detected by NGS	Dysembryoplastic neuroepithelial tumor	1%	No residual/recurrent disease at postoperative year 3
13	21 / M	Parietal lobe	Right parietal cortical, non-enhancing T2/FLAIR-hyperintense lesion	Dysplasia	Small monotonous round nuclei, cortical infiltrative pattern	GFAP, ATRX	SLC44A1::PRKCA alteration detected	Low-grade glioneuronal tumor	1%	No residual/recurrent disease at postoperative year 3
14	31 / F	Intraventricular region	Heterogeneous intraventricular mass involving both lateral ventricles; radiological impression favored central neurocytoma	Central neurocytoma	Small monotonous round nuclei, perinuclear halos, cortical infiltrative pattern	TTF1, synaptophysin	No pathogenic alteration detected by NGS	Central neurocytoma	4%	No residual/recurrent disease at postoperative year 2
15	55 / F	Frontal lobe	Large left frontal infiltrative mass involving corpus callosum/septum with cystic areas, diffusion restriction, and extensive edema	High-grade glial tumor	Small monotonous round nuclei, perinuclear halos, calcification, chicken-wire capillaries, initial oligodendroglioma consideration, cortical infiltrative pattern	GFAP, Olig2, p53	TERT promoter mutation detected; no IDH mutation; no 1p/19q codeletion	Glioblastoma, IDH-wildtype, molecularly defined	10 %	Died at postoperative year 1

Case	Age / sex	Location	Radiological summary	Initial microscopic impression	Oligodendroglioma-like features	Positive IHC markers	Molecular / FISH / NGS findings	Final integrated diagnosis	Ki-67	Follow-up
16	33 / F	Intraventricular region	Large heterogeneously enhancing mass involving both lateral ventricles with extensive calcification	Ependymoma	Small monotonous round nuclei, perinuclear halos	TTF1, synaptophysin	No pathogenic alteration detected by NGS	Central neurocytoma	5%	No residual/recurrent disease at postoperative year 2
17	26 / F	Lateral ventricle	Left lateral ventricular heterogeneous mass with coarse calcification; radiological differential included choroid plexus papilloma	Ependymoma	Small monotonous round nuclei, perinuclear halos, chicken-wire capillaries, microcystic background	TTF1, synaptophysin	No pathogenic alteration detected by NGS	Central neurocytoma	8%	No residual/recurrent disease at postoperative year 1
18	7 / M	Parietal lobe	Right parietal lobulated mass with cystic-necrotic areas, calcification, hemorrhagic foci, and edema	Astroblastoma	Small monotonous round nuclei, perinuclear halos, chicken-wire capillaries, cortical infiltrative pattern	GFAP, D2-40, p53	ZFTA alteration detected by NGS	Supratentorial ependymoma	15 %	No residual/recurrent disease at postoperative year 2
19	24 / F	Frontal lobe	Right frontal peripherally enhancing mass with surrounding edema	Astroblastoma	Small monotonous round nuclei, perinuclear halos	GFAP, Olig2, synaptophysin	PRKCA fusion detected by NGS	Low-grade glioneuronal tumor	1%	No residual/recurrent disease at postoperative year 1
20	53 / F	Frontal lobe	Left frontal heterogeneous enhancing lobulated mass extending toward periventricular white matter, cingulate gyrus, and corpus callosum with edema	Oligodendroglioma	Small monotonous round nuclei, perinuclear halos, calcification, cortical infiltrative pattern	GFAP, Olig2, IDH1 R132H	IDH mutation detected by NGS; no 1p/19q codeletion by FISH	Diffuse astrocytoma, IDH-mutant, CNS WHO grade 2	4%	Recurrence at postoperative month 18
21	41 / M	Frontal lobe	Left frontotemporal centrally cystic-necrotic mass with peripheral enhancement and extensive edema	Metastatic epithelial tumor	Small monotonous round nuclei, perinuclear halos, chicken-wire capillaries, cortical infiltrative pattern	PAX8, vimentin, PAX2, CD10	No pathogenic alteration detected by NGS; no 1p/19q codeletion by FISH	Metastatic renal cell carcinoma	5%	Recurrence at postoperative year 1
22	17 / F	Frontal lobe	Left superior frontal lesion with small juxtacortical cystic areas, foamy appearance, mild FLAIR hyperintensity, cortical irregularity, and hemosiderin-like rim	DNET	Small monotonous round nuclei, perinuclear halos, microcystic background	Olig2, NeuN, S100	No pathogenic alteration detected by NGS; no 1p/19q codeletion by FISH	Dysembryoplastic neuroepithelial tumor	1%	No residual/recurrent disease at postoperative year 1
23	26 / M	Temporal lobe	Right temporal subcortical cystic-hemorrhagic mass lesion measuring 2.4 ×	Pilocytic astrocytoma / low-grade glial tumor	Focal oligodendroglioma-like areas with monotonous round nuclei,	GFAP, Olig2, SOX10	KIAA1549::BRAF fusion detected by NGS; no IDH mutation detected by	Pilocytic astrocytoma, KIAA1549::BRAF fusion-positive	4%	No residual tumor postoperatively; no evidence of recurrence on

Case	Age / sex	Location	Radiological summary	Initial microscopic impression	Oligodendroglioma-like features	Positive IHC markers	Molecular / FISH / NGS findings	Final integrated diagnosis	Ki-67	Follow-up
			2.2 cm; no postoperative residual tumor		perinuclear halos, and calcification		NGS; no 1p/19q codeletion by FISH			follow-up MRI at postoperative year 2

Abbreviations: CD10, cluster of differentiation 10; CNS, central nervous system; DNET, dysembryoplastic neuroepithelial tumor; F, female; FCD, focal cortical dysplasia; FISH, fluorescence in situ hybridization; GFAP, glial fibrillary acidic protein; IDH, isocitrate dehydrogenase; IHC, immunohistochemistry; M, male; NGS, next-generation sequencing; Olig2, oligodendrocyte transcription factor 2; PA, pilocytic astrocytoma; PLNTY, polymorphous low-grade neuroepithelial tumor of the young; SOX10, SRY-box transcription factor 10.
