# Supplementary material for: Diagnostic Pitfalls of Oligodendroglioma-like Morphology: Integrated Reappraisal of 23 Non-Oligodendroglial Central Nervous System Tumors
Source: Diagnostics (Basel). 2026 Aug 26;16(17):2723. doi: 10.3390/diagnostics16172723 (PMC13564585; doi:10.3390/diagnostics16172723)
Supplement: Supplementary file 1 [file diagnostics-16-02723-s001.zip › Supplementary Table S2.pdf]

Supplementary Table S2. Diagnostic evidence excluding oligodendroglioma and supporting final integrated diagnoses

Final diagnosis/group	Evidence excluding oligodendroglioma	Positive evidence supporting final diagnosis	CIC/FUBP1 status	Methylation profiling
Astrocytoma, IDH-mutant	IDH mutation with absence of 1p/19q codeletion	ATRX loss/p53 expression pattern and astrocytic immunophenotype	Not systematically assessed	Not systematically performed
Glioblastoma, IDH-wildtype, CNS WHO grade 4	IDH-wildtype status and absence of 1p/19q codeletion	*TERT* promoter mutation and high-grade diffuse glioma context	Not systematically assessed	Not systematically performed
DNET / low-grade glioneuronal tumor	Lack of oligodendroglioma-defining integrated profile	Cortical/microcystic architecture, neuronal elements, NeuN/synaptophysin, *PRKCA* alteration when present	Not systematically assessed	Not systematically performed
PLNTY	Lack of oligodendroglioma-defining integrated profile	CD34 expression, low-grade neuroepithelial tumor context, calcification	Not systematically assessed	Not systematically performed
Central neurocytoma	Lack of oligodendroglioma-defining integrated profile	Intraventricular location and neurocytic immunophenotype	Not systematically assessed	Not systematically performed
Supratentorial ependymoma	Lack of oligodendroglioma-defining integrated profile	Ependymal morphology, GFAP/EMA pattern, *ZFTA* alteration	Not systematically assessed	Not systematically performed
Metastatic renal cell carcinoma	Lack of primary glioma profile	PAX8/PAX2/CD10/vimentin and epithelial marker expression	Not applicable/not systematically assessed	Not performed
Pilocytic astrocytoma	IDH-wildtype status and absence of 1p/19q codeletion	Biphasic piloid morphology, Rosenthal fibers/eosinophilic granular bodies, *KIAA1549::BRAF* fusion	Not systematically assessed	Not systematically performed
